# Eusociality outcompetes egalitarian and solitary strategies when resources are limited and reproduction is costly

**DOI:** 10.1002/ece3.4737

**Published:** 2018-12-11

**Authors:** Emanuel A. Fronhofer, Jürgen Liebig, Oliver Mitesser, Hans Joachim Poethke

**Affiliations:** ^1^ Department of Aquatic Ecology Eawag: Swiss Federal Institute of Aquatic Science and Technology Dübendorf Switzerland; ^2^ Department of Evolutionary Biology and Environmental Studies University of Zurich Zürich Switzerland; ^3^ ISEM Université de Montpellier CNRS, IRD, EPHE Montpellier France; ^4^ Field Station Fabrikschleichach University of Würzburg Rauhenebrach Germany; ^5^ School of Life Sciences and Center for Social Dynamics and Complexity Arizona State University Tempe Arizona

**Keywords:** cooperation, resource sharing, risk‐sensitive foraging, sociality, supersaturation

## Abstract

Explaining the evolution and maintenance of animal groups remains a challenge. Surprisingly, fundamental ecological factors, such as resource variance and competition for limited resources, tend to be ignored in models of cooperation. We use a mathematical model previously developed to quantify the influence of different group sizes on resource use efficiency in egalitarian groups and extend its scope to groups with severe reproductive skew (eusocial groups). Accounting for resource limitation, the model allows calculation of optimal group sizes (highest resource use efficiency) and equilibrium population sizes in egalitarian as well as eusocial groups for a broad spectrum of environmental conditions (variance of resource supply). We show that, in contrast to egalitarian groups, eusocial groups may not only reduce variance in resource supply for survival, thus reducing the risk of starvation, they may also increase variance in resource supply for reproduction. The latter effect allows reproduction even in situations when resources are scarce. These two facets of eusocial groups, resource sharing for survival and resource pooling for reproduction, constitute two beneficial mechanisms of group formation. In a majority of environmental situations, these two benefits of eusociality increase resource use efficiency and lead to supersaturation—a strong increase in carrying capacity. The increase in resource use efficiency provides indirect benefits to group members even for low intra‐group relatedness and may represent one potential explanation for the evolution and especially the maintenance of eusociality and cooperative breeding.

## 1 INTRODUCTION

The evolution and maintenance of cooperative behavior in animals has been a topic of ongoing interest since the days of Darwin. A number of possible factors favoring cooperation have been proposed (reviewed in Krause & Ruxton, 2002; Nowak, 2006; Lehmann & Keller, 2006; West, Griffin, & Gardner, 2007) including direct benefits of cooperation and indirect benefits that are received through increased fitness of relatives (Hamilton, 1964a,b), for instance. We focus on ecological factors and constraints (see e.g., Avila & Fromhage, 2015) as a driving force for the evolution and maintenance of cooperative behavior, specifically resource variance and competition for limited resources.

**Figure 1:**
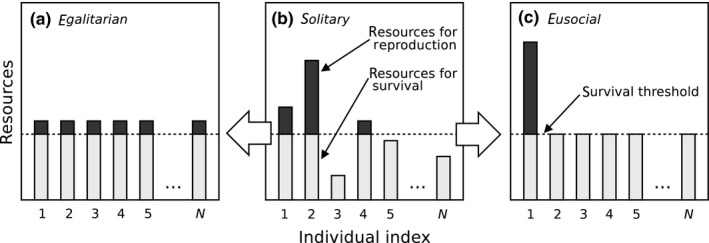
Schematic comparison of different modes of resource sharing in groups of animals. Individuals collect resources individually and vary in their success over one reproductive period (i.e., a season). Solitary individuals (b) thus differ in the amount of resources individuals may use for survival (light gray) and reproduction (dark gray). Some individuals (3, 5, and *N*) will die of starvation and not reproduce because they are not able to cross the survival threshold (dashed line). In egalitarian groups (a), resources are evenly shared after solitary foraging (b). All individuals can survive and receive a small amount for reproduction. In eusocial groups (c), individuals receive sufficient resources for survival and channel all remaining resources to the reproductive dominant individual (here individual 1). Of course, the evolutionary advantages of these resource sharing strategies will be impacted by how resources are exactly translated into survival and offspring, that is, the mortality and fertility functions, which is beyond the scope of this schematic representation. Note that the term “eusocial” group in this context only implies that only one individual will reproduce

Food resources, specifically the mean amount of resources as well as resource variance, affect foraging decisions as suggested by risk‐sensitive foraging theory (reviewed in Bednekoff, 1996; Kacelnik & Bateson, 1996; Smallwood, 1996; Bateson, 2002). In this context, group formation has traditionally been seen as a risk‐averse mechanism reducing the variance in resource supply (Caraco, 1981; Caraco, Uetz, Gillespie, & Giraldeau, 1995; Clark & Mangel, 1986; Uetz, 1996; Uetz & Hieber, 1997; Wenzel & Pickering, 1991). The simple idea behind these models is that foraging success may vary: individuals may find more resources than what they are able to fully utilize, or alternatively, they may not find any resources at all, which leads to certain death (Figure 1b). Foraging with subsequent egalitarian resource sharing in groups allows animals to dampen such environmental variance (Figure 1a), as all group members will receive an intermediate amount of resources which may guarantee survival and reproduction (see also Fronhofer, Pasurka, Mitesser, & Poethke, 2011).

Yet, as Poethke and Liebig (2008) point out, group formation is not necessarily a variance‐reducing mechanism. It may be seen as an important means of variance manipulation in general: whether variance in resource availability is reduced or increased depends on the degree of reproductive division of labor. While egalitarian resource allocation decreases intra‐group variance as explained above (Figure 1a), skewed resource allocation, by contrast, increases variance (Figure 1c). If resource availability and variance are low, solitary foragers may collect more resources than needed for survival, but not enough to reproduce in one reproductive period. If individuals forage, subsequently pool the surplus of resources not needed for survival and then redirect this surplus toward one (or a few) individual(s), individuals in such groups will survive and specific group members have a chance to reproduce. Throughout our analysis, we will use the term “eusocial” for highly skewed reproduction with one reproductive individual only. Depending on the shape of the fertility function such skewed reproduction clearly may increase fitness, either through direct fitness benefits (for the reproductive individual) or through indirect fitness benefits (via intra‐group relatedness). Thus, Poethke and Liebig (2008) suggest that egalitarian groups, as a risk‐reducing foraging strategy, should be favored in environments with high resource variance and eusocial animal groups should be favored in habitats with low resource variance, since this group structure increases inter‐individual variance.

Yet, in nature, egalitarian animal groups are only rarely found (Packer, Pusey, & Eberly, 2001). We assume that this discrepancy between model predictions and empirical observations stems from the fact that previous theoretical work on eusocial group formation as a risk‐sensitive foraging strategy accounts for a limited individual foraging rate (including Poethke & Liebig, 2008) but ignores the feedback of competition for limited resources, that is, the interaction between population size and resource availability. More concretely, while the relevant models may assume foraging success to be a function of forager strategies, foraging success is usually not modeled as being a function of the emerging number of individuals, that is, competition for limited resources which leads to density dependence at the population level. However, competition for resources has been shown to be of high relevance in the context of risk‐sensitive foraging in general (Fronhofer, Pasurka, Poitrineau, Mitesser, & Poethke, 2011) and for egalitarian resource sharing in particular (Fronhofer, Pasurka, Mitesser, et al., 2011). The latter work clearly demonstrates that including resource limitation into models of egalitarian resource sharing yields a more complex evolutionary pattern than the simple dichotomy of risk‐prone or risk‐averse behavior.

In the following we will extend the work of Fronhofer, Pasurka, Mitesser, et al. (2011), which focuses on egalitarian resource sharing, to groups with reproductive skew. We will thus compare two types of cooperative animal groups: On the one hand, individuals forming egalitarian groups forage and subsequently share the pooled resources so that every group member receives roughly the same amount of resources (examples include lions and social spiders: Packer et al., 2001; Whitehouse & Lubin, 2005). On the other hand, one can find animal groups in which just one individual receives all the resources for reproduction while the other members of the group only obtain a share necessary for their survival (Wilson, 1971; Clutton‐Brock, West, Ratnieks, & Foley, 2009, for example, eusocial insects or mole‐rats). Such groups are henceforth termed “eusocial.” The term “despotic,” which one may also find in the literature, is equivalent here. Of course, egalitarian and eusocial groups are rare situations at the ends of a continuum of different degrees of reproductive division of labor (that is, skew, for a review see Reeve & Keller, 2001). Evidently, as Sherman, Lacey, Reeve, and Keller (1995) point out, other degrees of reproductive division of labor in between these two extremes are possible and often encountered (for numerous examples from insect societies alone see Wilson, 1971; Hölldobler & Wilson, 1990; Costa, 2006; Hölldobler & Wilson, 2009). Nevertheless, theory suggests that these extremes may be favored over intermediate strategies (Cooper & West, 2018). We will here focus on the extremes for simplicity.

We will calculate the amount of resources required by groups across different group sizes in population equilibrium. This allows us to compare the competitive ability of the different strategies (type *T* ∊ {“*egalitarian*”, “*eusocial*”} and size of group *N* ∊ {1, 2, 3, …}) and to determine optimum group sizes for egalitarian and eusocial groups by identifying the group sizes that are most competitive, that is, minimize the amount of resources required at equilibrium. We find that, in contrast to egalitarian groups, eusocial groups may not only reduce variance in resource supply for survival, thus reducing the risk of starvation, they may also increase variance in resource supply for reproduction within the group. The latter effect allows reproduction even in situations when resources are scarce, which gives eusocial groups a competitive advantage over egalitarian groups and solitary strategies, specifically when resources are limiting and reproduction is costly. At the population level, this competitive advantage leads to increased carrying capacities, a phenomenon which has been termed “supersaturation” in cooperatively breeding birds (Dickinson & Hatchwell, 2004).

## 2 MODEL DESCRIPTION AND NUMERICAL RESULTS

### 2.1 Resource availability

We assume stochastic foraging, that is, individual foraging success follows a random distribution and the per capita probability of collecting an amount *x* of resources during one reproductive period is given by a probability density function $P(x,\overset{¯}{x},\theta )$. For the sake of simplicity, we assume that individuals collect resource items of limited size. Thus variance in foraging success is determined by a mean resource item size *θ* and a distribution of resources can easily be described by a Gamma distribution:

(1) $$P\left(x,\overset{¯}{x},\theta \right)={\left(\frac{x}{\theta }\right)}^{\frac{\overset{¯}{x}}{\theta }}\frac{e^{-\frac{x}{\theta }}}{x\Gamma \left(\frac{\overset{¯}{x}}{\theta }\right)}$$

with mean $\overset{¯}{x}$, scale parameter *θ*, and the gamma function Γ (Andrews, Askey, & Roy, 2001).

**Figure 2:**
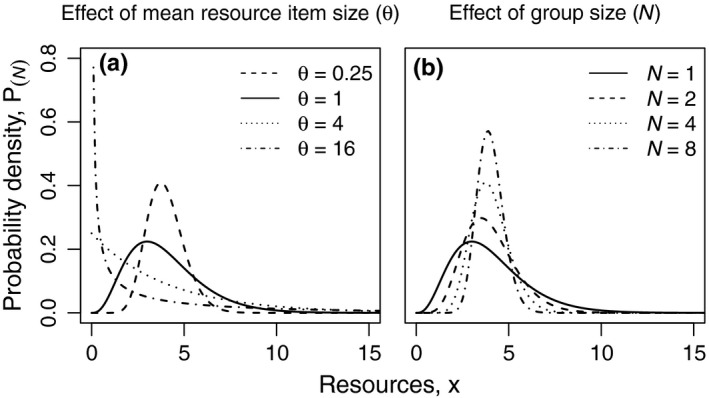
Distribution of resources (*x*) available to an individual. Influence of mean resource item size θ (a) and group size *N* (b) on the distribution of resources *x* available to an individual as a solitary $P(x,\overset{¯}{x},\theta )$ (Equation (1)) respectively $P_{N}\left(x,\overset{¯}{x},\theta \right)$ in a group of size *N* (Equation (2)). (a) $\overset{¯}{x}=4$; *N* = 1; θ = 0.25 (dashed line), θ = 1 (solid line), θ = 4 (dotted line), θ = 16 (dotdashed line). (b) $\overset{¯}{x}=4$; θ = 1; *N* = 1 (solid line), *N* = 2 (dashed line), *N* = 4 (dotted line), *N* = 8 (dotdashed line)

For integer ratios $\kappa =\overset{¯}{x}\theta^{-1}$, this Gamma distribution results from summing up κ independent, identical and exponentially distributed random variables with scale parameter *θ* (and thus mean *θ*). Such a random number can be interpreted as the size of an item collected during one of κ foraging trips of a single individual. The variance of acquired resources is $\sigma^{2}=\overset{¯}{x}\theta$ and the coefficient of variation $\text{CV}=\sqrt{\frac{\theta }{\overset{¯}{x}}}$. For a constant mean amount of resources $\overset{¯}{x}$ collected by an individual, an increase in item size will necessarily be accompanied by an increase in the variance of the amount of resources collected (Figure 2a). In the following, we will therefore use mean resource item size θ as a proxy for environmental variance.

For both individuals in egalitarian as well as individuals in eusocial groups, we assume that foraged resources are pooled and subsequently allocated to survival and reproduction. The process of resource pooling in a group of size *N* modifies the resource distribution. The summation of *N* Gamma‐distributed random variables with identical scale parameter yields another Gamma distribution with variance increased by a factor of *N*. Therefore, the calculation of the per capita resource distribution requires a subsequent division by *N* of the summed random variable and thus a division by *N* ^2^ of the variance. In total, variance in per capita resources decreases with 1/*N* and the amount of resources available per individual in a group of size *N* follows a modified Gamma distribution with reduced variance:

(2) $$P_{N}\left(x,\overset{¯}{x},\theta \right)={\left(\frac{\mathrm{Nx}}{\theta }\right)}^{\frac{N\overset{¯}{x}}{\theta }}\frac{e^{-\frac{\mathrm{Nx}}{\theta }}}{x\Gamma \left(\frac{N\overset{¯}{x}}{\theta }\right)}$$

### 2.2 Fertility and mortality

**Figure 3:**
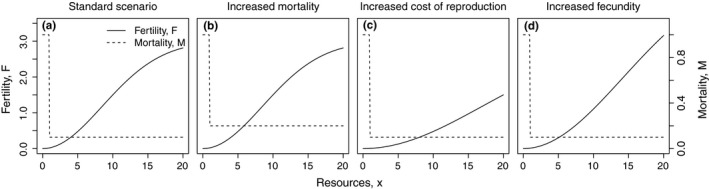
Influence of model parameters on the fertility function (*F*(*x* = *x*_*r*_); solid line) and the mortality function (*M*(*x* = *x*_*s*_); dashed line) for four exemplary parameter combinations. (a) standard parameter set (*F*_max_ = 3; *c*_0_ = 4; *M*_*b*_ = 0.1; *o*_*M*_ = 1); (b) increased mortality (*F*_max_ = 3; *c*_0_ = 4; *M*_*b*_ = 0.2; *o*_*M*_ = 1); (c) increased cost of reproduction (*F*_max_ = 3; *c*_0_ = 8; *M*_*b*_ = 0.1; *o*_*M*_ = 1); (d) increased fecundity (*F*_max_ = 5; *c*_0_ = 4; *M*_*b*_ = 0.1; *o*_*M*_ = 1)

We assume that individual mortality *M* is a function of the amount of resources *x*_*s*_ allocated to survival and, as a simplification, we use a step function. We therefore assume that an animal dies if it receives less resources than a certain threshold resource value (*o*_*M*_) and survives with probability (1 − *M*(*x*_*s*_)) if it receives more (Figure 3):

(3) $$M\left(x_{s}\right)=\left\{\begin{array}{cc} 1 & \text{if}\;x_{s}<o_{M}\\ M_{b} & \text{if}\;x_{s}\geq o_{M} \end{array}\right.$$

with the resource independent baseline mortality *M*_*b*_, resulting from predation or disease, for instance. We previously analyzed the influence of including a sigmoid function for mortality and could show that this does not change our results qualitatively (Fronhofer, Pasurka, Mitesser, et al., 2011).

Pooled resources are first allocated to survival of group members until all individuals have received the amount *x*_*s*_ = *o*_*M*_ preventing death from starvation. Individuals die if there are not sufficient resources available. We thus get for the per capita mortality in a group of size *N*:

(4) $$\mu \left(N,\overset{¯}{x},\theta \right)=\int_{0}^{\infty }P_{N}\left(x_{s},\overset{¯}{x},\theta \right)M\left(x_{s}\right)\text{d}x_{s}$$

All remaining resources are allocated to reproduction, either by giving them to a single reproductively dominant individual (eusocial groups) or by equally sharing them between all members of the group (egalitarian groups). In general, reproduction *F* is a function of the resources available per capita *x*. As fertility is not unlimited, the functional relationship between fertility and the resources remaining after consumption for survival *x*_*r*_ = max(0, *x* − *o*_*M*_) allocated to reproduction can be assumed to follow a sigmoid shape (Figure 3):

(5) $$F\left(x_{r}\right)=F_{\max}\left(1-e^{-{\left(\frac{x_{r}}{c_{0}F_{\max}}\right)}^{2}}\right)$$

where *F*_max_ determines fecundity, that is, the maximal value the reproduction function can take. For low values of *x*_*r*_, the steepness of the fertility function is determined by 1/*c*_0_. Therefore, *c*_0_ can be interpreted as the cost of reproduction. For an overview of parameter combinations under consideration see Table 1.

**Table 1:** Model parameters, meaning and tested values. Note that fecundity (*F*_max_) is a net rate, that is, for solitaries *F*_max_ = 5 leads to a quintupling of population size

Parameter	Values	Meaning
*F*_max_	[3, 5]	Fecundity, i.e., maximal number of offspring
*c*_0_	[4, 8]	Costs of offspring production
θ	]0, 32]	Environmental variance (mean item size)
*M*_*b*_	[0.1, 0.2]	Baseline mortality (resource independent)
*o*_*M*_	1.0	Minimum amount of resources needed for survival

Using Equation (5), we may calculate the per capita natality for individuals in groups of size *N* as a function of group size. For individuals in egalitarian groups one obtains

(6) $$\phi_{\mathrm{egalitarian}}\left(N,\overset{¯}{x},\theta \right)=\int_{0}^{\infty }P_{N}\left(x,\overset{¯}{x},\theta \right)F\left(x_{r}\right)\text{d}x$$

and for individuals in eusocial groups with only one reproductive individual

(7) $$\phi_{\mathrm{eusocial}}\left(N,\overset{¯}{x},\theta \right)=\frac{1}{N}\int_{0}^{\infty }P_{N}\left(x,\overset{¯}{x},\theta \right)F\left(Nx_{r}\right)\text{d}x$$

with *x*_*r*_ = max(0, *x* − *o*_*M*_). Note that expression 7 can be generated from expression 6 by shifting the evaluation of the fertility function to *N* times greater resource values and dividing by the number of individuals for calculation of the per capita rate.

Both per capita natality (Equations (6) and (7)) and mortality (Equation (4)) are functions of the distribution of resources acquired by individuals ($P_{N}\left(x,\overset{¯}{x},\theta \right)$, Equation (2)). While fertility increases with the amount of resources available to individuals, mean mortality decreases when resources become more abundant. We assume that resources are limited. Thus, resources available to individuals decrease with increasing population size and populations reach their equilibrium population size (“carrying capacity”) when mortality balances natality. We may thus formulate the equilibrium condition for populations consisting of individuals in groups of size *N* as

(8) $$\phi \left(N,{\overset{¯}{x}}_{N},\theta \right)=\mu \left(N,{\overset{¯}{x}}_{N},\theta \right).$$

**Figure 4:**
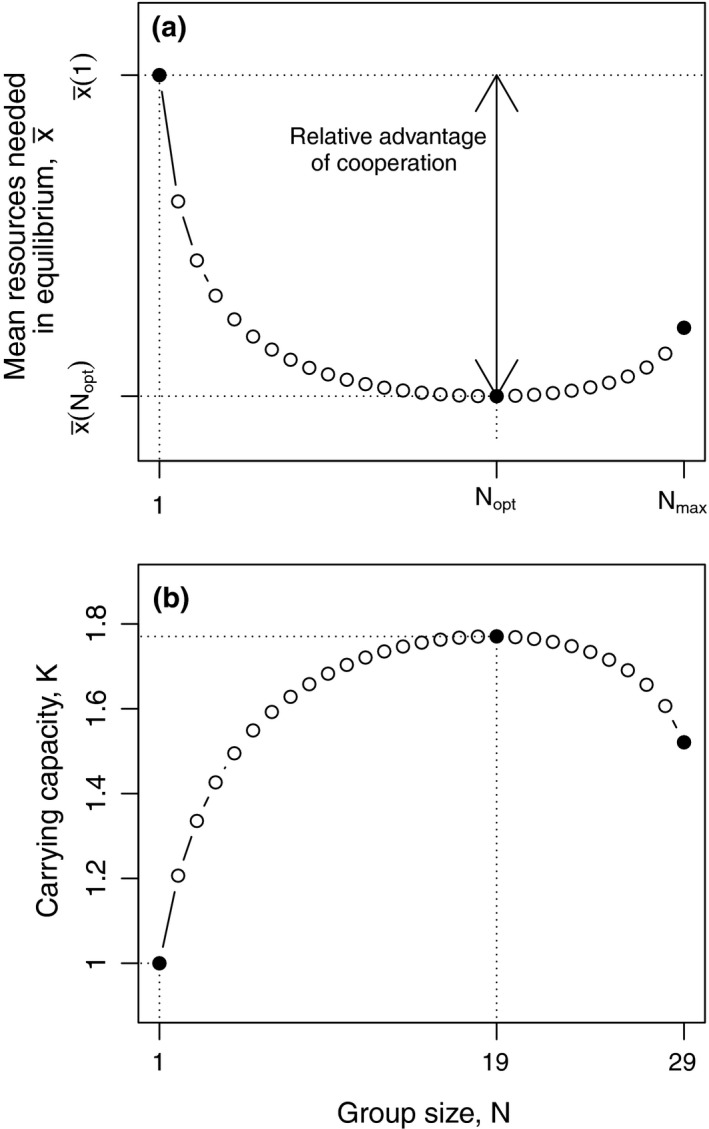
Influence of group size on (a) mean amount of resources needed to balance mortality and fertility in eusocial groups and (b) corresponding carrying capacity. Carrying capacity is shown relative to the carrying capacity of the solitary strategy. Numerical solution of Equation (8) for *F*_max_ = 3, *c*_0_ = 4, *M*_*b*_ = 0.1, θ = 5 and *o*_*M*_ = 1

Equation (8) yields an implicit relation that allows us to determine the influence of group size *N* and resource item size *θ* on the minimal mean amount of resources ${\overset{¯}{x}}_{N}$ per individual needed to balance reproduction and mortality (Figure 4a). The carrying capacity is then determined by the total amount of resources available (*X*) and the minimal amount of resources required per individual ${\overset{¯}{x}}_{N}$:

(9) $$K\left(N\right)=\frac{X}{{\overset{¯}{x}}_{N}}.$$

If current total population size *N*_tot_ is less than *K*, the mean per capita amount of resources available for individuals ($\overset{¯}{x}$) is greater than ${\overset{¯}{x}}_{N}$ resulting in an increase in population size as natality is greater than mortality. The opposite is the case if *N*_tot_ > *K*.

We assume that the evolution of an optimal grouping strategy (group size and type) will increase resource use efficiency, thus minimizing resource requirement in equilibrium and maximizing carrying capacity (see among others MacArthur, 1962; MacArthur & Wilson, 1967; Boyce, 1984; Lande, Engen, & Saether, 2009). A minimization of ${\overset{¯}{x}}_{N}$ (Figure 4) thus allows us to determine the optimal group size *N*_opt_ (Fronhofer, Pasurka, Mitesser, et al., 2011; Fronhofer, Pasurka, Poitrineau, et al., 2011). Clearly, it is well known that evolution does not generally maximize carrying capacity (e.g., Fronhofer & Altermatt, 2015; Fronhofer, Nitsche, & Altermatt, 2017; Matessi & Gatto, 1984; Reznick, Bryant, & Bashey, 2002). In order to show that, under the model assumptions outlined above, optimal strategies that maximize carrying capacity are indeed continuously stable strategies, we compare the results of our optimality approach with an invasibility analysis in the Appendix S1.

Strictly speaking, our reasoning only holds if all group members have the possibility to reproduce, which, of course, is given in egalitarian groups and holds for eusocial groups if the reproductive individual is determined by a lottery. However, in eusocial groups, subordinates may never be able to reproduce. In the latter case, the optimal group size derived as described above may not be evolutionarily stable, as subordinates will mainly benefit from indirect fitness gains via relatedness. This implies that we have to take into account the degree of intra‐group relatedness. For simplicity, we will first present results that hold true if the reproductive individual of eusocial groups is defined by a lottery or if intra‐group relatedness equals 1. We will then relax this assumption, introduce intra‐group relatedness <1 into our model and explore its robustness in the section “Joining or leaving a group: evolutionary stability of eusocial groups” below.

### 2.3 Optimal group sizes and minimum resource requirements

**Figure 5:**
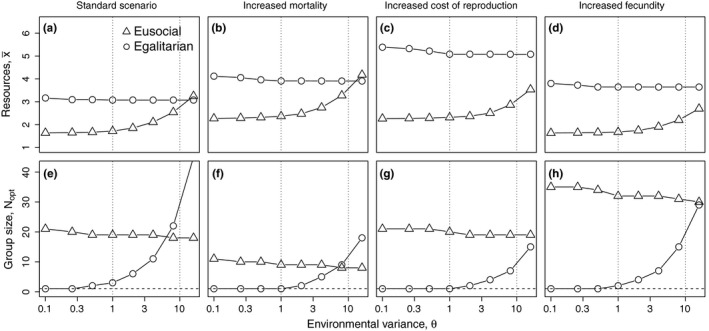
Influence of environmental variance (θ) on mean amount of resources needed ($\overset{¯}{x}$, upper row) and optimal group size (*N*_opt_, lower row) for four exemplary parameter combinations (see Figure 3). (a, e) standard parameter set (*F*_max_ = 3; *c*_0_ = 4; *M*_*b*_ = 0.1; *o*_*M*_ = 1); (b, f) increased mortality (*F*_max_ = 3; *c*_0_ = 4; *M*_*b*_ = 0.2; *o*_*M*_ = 1); (c, g) increased cost of reproduction (*F*_max_ = 3; *c*_0_ = 8; *M*_*b*_ = 0.1; *o*_*M*_ = 1); (d, h) increased fecundity (*F*_max_ = 5; *c*_0_ = 4; *M*_*b*_ = 0.1; *o*_*M*_ = 1). Circles give the results for egalitarian, triangles those for eusocial groups. Note the logarithmic *x*‐axis. The vertical dotted lines refer to the three different cases discussed in the main text

As Equation (8) cannot be solved analytically, we approximated the results numerically. Figure 5 gives the resulting mean amount of resources needed at population equilibrium and the correspondent optimal group sizes for a broad range of environmental variance (0.1 ≤ *θ* ≤ 16). For a mean amount of resources collected per individual of approximately $\overset{¯}{x}\approx 4$, this corresponds to a coefficient of variation ranging from CV ≈ 0.16 to CV ≈ 2 (see Equations (1) and Figure 2). The results reveal that reproductive skew in eusocial groups experiencing competition does not simply increase inter‐individual variance, and the benefit of this strategy is not restricted to situations of low environmental variance, as postulated by Poethke and Liebig (2008).

A careful analysis of the results reveals that we must distinguish three fundamentally different situations: Firstly, for very low values of environmental variance (approx. *θ* ≤ 1; Figure 5, left dotted line) a reduction of variance would actually decrease the expected fitness of individuals. This is due to the fact that under low resource availability (which is implied in our population equilibrium assumption; Equation (8)) the fertility function is convex (Figure 3) and Jensen's inequality (Ruel & Ayres, 1999) predicts a decrease in mean reproductive success for decreased variance in resource supply. Thus, solitary strategies will out‐compete egalitarian groups (that is, optimal group sizes of egalitarian groups become *N*_opt_ = 1; see Figure 5e–h) in this area of parameter space. Eusocial groups, on the other hand, can increase inter‐individual variance in resource supply and will consequently out‐compete solitary strategies (Figure 5a–d).

Secondly, for intermediate values of environmental variance (approx. 1 < *θ* ≤ 10; Figure 5, between the two dotted line) variance reduction is obviously beneficial, which can be seen from the increase in optimal group sizes of egalitarian groups with increasing environmental variance (Figure 5e–h). Nevertheless, eusocial groups perform better than egalitarian groups in this range of environmental variances (*θ*). This is readily explained by the differential effect of inter‐individual variance in resource supply on fertility on the one hand and mortality on the other. In this range of *θ* values, a reduction of variance reduces the mortality risk of individuals. However, it also reduces mean reproductive success. As long as the former effect dominates, it still pays off for egalitarian groups to reduce variance. However, eusocial groups may use both mechanisms to increase their performance. They reduce inter‐individual variance in resource supply for survival and, at the same time, increase variance in resource supply for reproduction. This combination of two beneficial effects of eusocial group formation, which has not been highlighted in previous work, explains the success of eusocial groups under a wide range of intermediate variance in foraging success.

Thirdly, if environmental variance increases even further (approx. *θ* ≥ 10; Figure 5, right dotted line), an additional increase of inter‐individual variance in resource supply for reproduction by channeling all resources to a reproductive dominant individual is no longer profitable. Consequently, variance reduction by forming egalitarian groups may become the superior strategy, depending on the other parameters (Figure 5a,b).

Clearly, these three phases result from our choice of a sigmoid fertility function (Equation (5)) which exhibits both convex and concave parts. Our fundamental reasoning based on Jensen's inequality (Ruel & Ayres, 1999) of course also applies for other shapes of fertility functions. However, specifically functions that are purely convex or concave will modulate results: for instance, increased convexity will increase the potential benefit of eusocial groups, as pointed out above.

### 2.4 The effect of fecundity and mortality

The success of eusocial groups depends on the ability of the reproductive individual to effectively use the resources it receives from members of the group. This ability is, however, critically limited by the maximum reproductive capacity *F*_max_ which limits the amount of baseline mortality that can be compensated by reproduction. Thus, at population equilibrium, the group size of eusocial groups is limited to $N_{\max}\leq \frac{F_{\max}}{M_{b}}$. It increases with increasing fecundity *F*_max_ (compare Figure 5h to e) and decreases with increasing mortality *M*_*b*_ (compare Figure 5f to e). *F*_max_ also influences the shape of the fertility function (*F*; Equation (5)) and larger values of *F*_max_ enlarge the convex part of this curve (Figure 3d). This increases the potential benefit eusocial groups can gain from increased inter‐individual variance in resources for reproduction. Consequently, in eusocial groups, the amount of resources needed at population equilibrium decreases and optimal group sizes severely increase with increasing values of *F*_max_ (compare Figure 5d to a). A similar argument holds for increased cost of reproduction *c*_0_. *c*_0_ also increases the convex part of the fertility function (Figure 3c) and consequently increases the benefit of eusocial groups (Figure 5c).

For egalitarian groups, the influence of *F*_max_ on resources needed at population equilibrium as well as on optimal group sizes is far less pronounced. As larger values of *F*_max_ enlarge the convex part of the fertility function and intra‐specific competition reduces the amount of resources available for reproduction *x*_*r*_, the variance reducing effect of egalitarian resource sharing actually reduces mean fertility $\phi (N,\overset{¯}{x},\theta )$. Thus, the amount of resources $\overset{¯}{x}$ needed by egalitarian groups increases (compare Figure 5d to a) and group sizes of egalitarian groups decrease (compare Figure 5h–e) with increasing fecundity *F*_max_.

Baseline mortality *M*_*b*_ has a similar effect on resource requirement. It increases the amount of resources $\overset{¯}{x}$ needed because higher baseline mortality must (at equilibrium) be compensated by higher reproduction and consequently by higher mean amounts of resources acquired. By contrast, costs of reproduction *c*_0_ have only a negligible effect on the amount of resources needed $\overset{¯}{x}$ as well as on optimal group sizes *N*_opt_ of egalitarian groups.

### 2.5 Joining or leaving a group: evolutionary stability of eusocial groups

For all results presented above, we assume that, whenever a large population of groups of size *N*_pop_ > 1 utilizes resources more efficiently (that is, reaches a higher carrying capacity) than a population of solitary individuals it can, in principle, not be invaded by individuals following a solitary strategy. This phenomenon is known from cooperatively breeding birds as “supersaturation” (Dickinson & Hatchwell, 2004). The group strategy of size *N*_pop_ > 1 evolves because, at population equilibrium, the groups would drive mean resource availability below the critical value that allows the growth of a solitary strategy.

As pointed out above, our results hold true for egalitarian groups in general and for eusocial groups if the reproductive individual is determined by a lottery or if intra‐group relatedness equals 1. However, in eusocial groups, the reproductive individual is often not determined by a lottery and intra‐group relatedness is likely <1. Therefore, direct fitness of subordinates is zero and the advantage of subordinates living in a eusocial group is solely determined by indirect fitness benefits, that is, by its relatedness to the offspring of the dominant individual (Hamilton, 1964a,b), if we ignore other direct benefits such as queuing for a dominant position (see, for example, Kokko & Johnstone, 1999). Thus, it would clearly be beneficial to leave a group of size *N*_pop_, and such groups would become unstable, if the fitness of a solitary individual in a population of groups of size *N*_pop_ exceeds the (inclusive) fitness of a subordinate in a group of size *N*_pop_.

To analyze the evolutionary stability of different strategies (that is, different group sizes *N*) in a heuristic extension of our model, we use a simple fitness measure: the lifetime reproductive success of an individual (Ψ). For our model, lifetime reproductive success may be derived from mean fertility $\phi (N,\overset{¯}{x},\theta )$ (Equation (7)) and the mean lifetime $\frac{1}{\mu (N,\overset{¯}{x},\theta )}$ (according to Equation (4)) of individuals as $\Psi =\frac{\phi (N,\overset{¯}{x},\theta )}{\mu (N,\overset{¯}{x},\theta )}$. Ψ is a function of the size *N* of the group an individual is a member of, the mean size of resource items *θ* collected by individuals and the mean amount of resources collected $\overset{¯}{x}$. As the latter is itself an emergent property resulting from intra‐specific competition in an equilibrium population of groups of size *N*_pop_, we may denote it as Ψ(*N*, *N*_pop_, θ). In a habitat saturated by groups with a specific group size *N*_pop_ = *N*, the rate of increase of groups of the same size will be Ψ(*N*, *N*, θ) = 1 at equilibrium, while it may take different values Ψ(*N*_*i*_, *N*, θ) ≠ 1 for any other group size *N*_*i*_ when *N* ≠ *N*_*i*_.

So far, we have used Ψ as the mean fitness of a strategy. However, in eusocial groups, the inclusive fitness Φ of an individual depends on its role. The inclusive fitness Φ_sub_(*N*, *N*, *θ*) of subordinates in a group of size *N* living in an infinitely large population of groups of the same size *N* is determined by their life expectancy $\frac{1}{\mu (N,{\overset{¯}{x}}_{N},\theta )}$ and the fertility $N\cdot \phi (N,{\overset{¯}{x}}_{N},\theta )$ of the related dominant as

(10) $$\Phi_{\mathrm{sub}}\left(N,N,\theta \right)=r\frac{N\phi (N,{\overset{¯}{x}}_{N},\theta )}{\mu (N,{\overset{¯}{x}}_{N},\theta )}$$

where *r* denotes the coefficient of relatedness while the mean amount of resources collected per individual ${\overset{¯}{x}}_{N}$ is a function of the population strategy *N*. When subordinates defect, leave the group and live as solitary individuals (*N*_*i*_ = 1) they will lose indirect fitness benefits (as the related group now lacks one subordinate helper) but gain direct fitness benefits as a reproducing solitary individual. Now their inclusive fitness will be

(11) $$\Phi_{\mathrm{def}}\left(1,N,\theta \right)=\frac{\phi (1,{\overset{¯}{x}}_{N},\theta )}{\mu (1,{\overset{¯}{x}}_{N},\theta )}+r\frac{\left(N-1\right)\phi \left(N-1,{\overset{¯}{x}}_{N},\theta \right)}{\mu (N-1,{\overset{¯}{x}}_{N},\theta )}.$$

Note that, strictly speaking, the inclusive fitness approach only holds as long as a strategy is not more likely to interact with itself than with unrelated strategies (for a detailed discussion see, for example, Hines & Maynard Smith, 1979). Subordinates should leave the group whenever leaving would result in a net increase in inclusive fitness, that is, when

(12) $$\Phi_{\mathrm{def}}\left(1,N,\theta \right)>\Phi_{\mathrm{sub}}\left(N,N,\theta \right).$$

**Figure 6:**
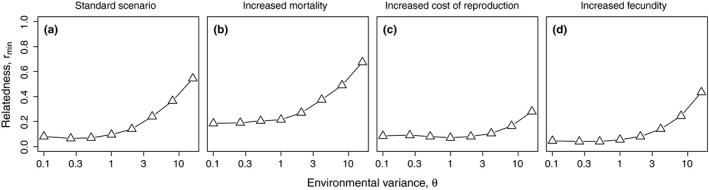
Influence of environmental variance (θ) on minimum relatedness *r*_min_ of group members required to secure stability of eusocial groups of optimal group size (*N*_opt_, see Figure 5e–h) for four exemplary parameter combinations. (a) standard parameter set (*F*_max_ = 3; *c*_0_ = 4; *M*_*b*_ = 0.1; *o*_*M*_ = 1); (b) increased mortality (*F*_max_ = 3; *c*_0_ = 4; *M*_*b*_ = 0.2; *o*_*M*_ = 1); (c) increased cost of reproduction (*F*_max_ = 3; *c*_0_ = 8; *M*_*b*_ = 0.1; *o*_*M*_ = 1); (d) increased fecundity (*F*_max_ = 5; *c*_0_ = 4; *M*_*b*_ = 0.1; *o*_*M*_ = 1). Note the logarithmic *x*‐axis

Equation (12) allows to derive the minimum relatedness *r*_min_ preventing individuals from leaving a group, that is, the minimum relatedness that allows the evolutionary stability of eusocial groups (see Figure 6).

Numerical solutions of Equation (12) (Figure 6) show that, particularly for low environmental variance θ, the benefit of eusocial groups is sufficient to make the role of subordinate group members attractive even for individuals only modestly related to the dominant (*r*_min_ ≈ 0.1). While increased baseline mortality *M*_*b*_ (Figure 6b) increases the relatedness necessary for the stability of eusocial groups, increased cost of reproduction *c*_0_ (Figure 6c) and increased fecundity *F*_max_ (Figure 6d) significantly decrease it and make eusocial groups evolutionarily stable even for extremely high environmental variance and low coefficients of relatedness (*r* < 0.25).

## 3 DISCUSSION

In contrast to the work of Poethke and Liebig (2008), the present model explicitly quantifies birth and death rates as functions of resource availability. This allows us to take into account competition for resources between individuals (see also Pen & Weissing, 2000) which reduces resource availability and ultimately results in selection for resource‐use efficiency. Our results show that, for a broad spectrum of model parameters, cooperative breeding with resource sharing, and in particular, the formation of eusocial groups with extreme reproductive skew, may substantially increase carrying capacity (Figure 5a–d). This will lead to the competitive exclusion of solitary foragers and breeders, a phenomenon known from cooperatively breeding birds as “supersaturation” (Dickinson & Hatchwell, 2004) which also makes a reversion to solitary breeding less likely.

Our results demonstrate the potential of variance manipulation as a driving force for the evolution of cooperative animal groups. It may thus have contributed to the evolution of eusocial animal groups. More importantly, the demonstrated ecological benefit of group formation may have been important for the stabilization of cooperative breeding or eusociality after the transition from solitary life had already occurred, as our model does not explicitly consider the initial mechanism of group formation. Our model provides an ecological explanation for the benefit of group formation which sets it apart from previous models of reproductive skew (Johnstone, 2000; Reeve & Keller, 2001; Vehrencamp, 1983) that are often based on a predefined arbitrary benefit of group formation. In our simple consumer‐resource model such an assumption is not required, as group formation evolves because of the emergent advantages of variance manipulation.

As mentioned earlier, Poethke and Liebig (2008) demonstrate that egalitarian group formation, a variance reducing foraging strategy, is favored at high resource variances and that, by contrast, eusocial groups or cooperative breeding is advantageous when resource variance is low, because this strategy increases inter‐individual variance in resource supply. However, when competition for resources is taken into account, as in the present study as a result of our population equilibrium assumption (Equation (8)), these predictions change. Eusocial groups remain at a clear advantage for low resource variances but become advantageous even for intermediate and rather high variance in resource availability (see Figure 5). This is due to the beneficial effects of eusocial groups on resource variance: (a) inter‐individual variance is indeed increased for reproduction, which makes reproduction possible even when solitary individuals do not collect sufficient resources for survival and reproduction. (b) At the same time, for survival the opposite is true, individuals that do not collect sufficient resources for survival as solitaries may survive in the group because they profit from resource sharing. The combined effect of these two mechanisms may explain the dominance of eusociality over egalitarian group.

### 3.1 Model limitations

Throughout this work, we have analyzed the formation of eusocial groups under equilibrium conditions. However, in a temporally and spatially heterogeneous landscape, and particularly in a metapopulation (Fronhofer, Kubisch, Hilker, Hovestadt, & Poethke, 2012), one will always find local populations that have not reached equilibrium density, yet. In newly colonized local habitat patches, for example, resources will usually be rather abundant and competition will be weak. This will necessarily favor solitary strategies with their high potential offspring numbers. Thus, landscape fragmentation and temporal heterogeneity in resource availability may lead to the coexistence of eusocial and solitary strategies.

While we do analyze the consequences of relatedness, and show that the ecological benefits of eusociality may be very large, which makes eusocial groups evolutionarily stable even at low levels of relatedness, our modeling procedure implicitly assumes that groups have already been formed and ignores the process of group formation. Group formation strategies are diverse and include the establishment of entire colonies after nest foundation by a single or few individuals as observed in halictid bees or wasps, for example, but also establishment after colony fission as in highly eusocial insect species like honeybees or ants. Our omission of the group formation process necessarily limits the scope of our analyses and highlights that our model may be best thought of as showcasing ecological benefits that are relevant for the maintenance and increase in size of already existing eusocial groups. Note that these restrictions do not apply to eusocial groups in which all members initially have a chance to become the dominant individual. Such groups can evolve by mutualism and indirect fitness benefits via relatedness are not necessary (see e.g., Rissing, Pollock, Higgins, Hagen, & Smith, 1989).

Furthermore, it is important to note that our inclusive fitness analysis is heuristic in the sense that we do not use an explicit model of evolutionary competition between different strategies. As Olejarz, Allen, Veller, and Nowak (2015) have shown recently, invasibility and stability of an altruistic allele need not be linear in any relatedness parameter and our analysis must therefore be seen as a conceptual extension of our model and not as an in‐depth analysis.

In addition to the points discussed with regards to relatedness, in both the egalitarian and eusocial case resource redistribution rules according to group type might be violated by cheating individuals which try to increase their reproductive share. However, this additional level of complexity is out of the scope of our approach and but has been analyzed elsewhere, for instance by Hamilton (2004), Wenseleers, Helanterä, Hart, and Ratnieks (2004), or Schneider and Bilde (2008).

A further limitation of our model is its comparison of only the two extreme cases of group formation (egalitarian vs. eusocial groups), while in nature one will observe a continuum of cooperative strategies (see, e.g., Sherman et al., 1995, but see Cooper & West, 2018). While this may impact our results quantitatively, the two beneficial mechanisms of eusocial group formation discussed above remain potentially important ecological mechanisms responsible for the evolution and maintenance of eusocial groups.

Of course, other factors (e.g., reviewed in Krause & Ruxton, 2002; Nowak, 2006; Lehmann & Keller, 2006) will also play a role for the evolution of eusociality and the relative importance of the different mechanisms may vary. Nevertheless, our model is general in the sense that dealing with limited resources and variance in resource supply are challenges likely faced by a majority of organisms.

Clearly, the ecological conditions we consider exclusively relate to the distribution and especially the variance in resource supply. While our model shows the relevance of intraspecific competition for resources, we do not consider interspecific competition or predation, for instance (see Rankin, López‐Sepulcre, Foster, & Kokko, 2007; Tsuji, 2013).

Finally, the assumption that evolution will minimize resource requirement and therefore maximize carrying capacity is valid for our model (see Appendix S1 for an invasibility analysis). However, this hinges upon our description of the resource distribution (Equation (1)) and the implicit assumption that the environmental resource distribution itself does not change over time (see Appendix S1). Therefore, our results are valid for consumers that feed on abiotic, renewing resources or for other consumer‐resource systems in which assimilation efficiency is maximized (see also Fronhofer & Altermatt, 2015; Matessi & Gatto, 1984; Reznick et al., 2002).

### 3.2 Empirical examples

It is interesting to note that, in our model, the increase in carrying capacity is generally more pronounced in eusocial than in egalitarian groups. Our model thus suggests that eusocial groups should dominate for a majority of environmental settings and life‐history strategies. Although our model is very simple and compares only the extreme cases of egalitarian and eusocial groups, the dominance of eusocial groups in nature can be observed empirically: most cooperative societies are eusocial while truly egalitarian groups seem to be rare (Packer et al., 2001).

Typical eusocial groups are found among insects. In accordance with our model, the ubiquitously present and very successful ants alone show a fascinating array of different life‐history strategies and feed on resources with typically low but also high variance (Hölldobler & Wilson, 1990). Interestingly, egalitarian societies have been reported from two ant species, *Ooceraea* (formerly *Cerapachys*) *biroi* (Tsuji & Yamauchi, 1995) and *Pristomyrmex punctatus* (Tsuji & Dobata, 2011), in which all workers reproduce and help others. While we can only speculate with regards to the evolutionary forces responsible for this secondarily evolved egalitarian behavior, *Pristomyrmex punctatus* shows rather low fecundities (Tsuji, 1988) and their nomadic life history may suggest important variance in resources, which is in line with our model predictions.

While these examples come from highly derived insect societies, our model may be more appropriate for primitively eusocial insects where subordinates are not sterile, for instance. An additional example are polistine wasps (reviewed in the context of skew theory in Reeve & Keller, 2001): While in the founding phase of a wasp nest the chance of becoming the reproductively dominant will make joining another female an attractive strategy, the probability to stay and accept the role of a “worker” will ultimately depend on the relatedness with the reproductively dominant individual. However, when an expensive nest is a prerequisite of successful reproduction this will change the shape of the fertility function. Such primary investments may be modeled as an offset that shifts the fertility function toward higher amounts of resources needed (Fronhofer, Pasurka, Mitesser, et al., 2011). Additional investments make reproduction more costly and will thus severely reduce the relatedness *r*_min_ (see Figure 6c) necessary to stabilize eusocial groups.

Cooperative systems with non‐reproductive helpers can also be found in cooperatively breeding birds (Dickinson & Hatchwell, 2004) and the phenomenon of “supersaturation” has been well described in his context. In line with our results that predict an advantage of eusocial groups at low baseline mortalities, Arnold and Owens (1998) report that cooperatively breeding birds that demonstrate some reproductive skew are generally characterized by low mortality rates. Furthermore, cooperative breeding seems to be consistently associated with low environmental variance in nature (Arnold & Owens, 1998, 1999; Ford, Bell, Nias, & Noske, 1988; Gonzalez, Sheldon, & Tobias, 2013), although Jetz and Rubenstein (2011) find evidence for the opposite pattern. Our model corroborates these findings as it predicts an advantage for cooperative breeding and eusocial groups for both low and high resource variance.

By contrast, eusocial societies are rare in mammals (Clutton‐Brock et al., 2009). Cooperative breeding with high reproductive skew or eusociality has only evolved in four taxa: marmosets and tamarins, dogs, diurnal mongooses and African mole‐rats. Typically, females in these groups show unusually high levels of fecundity.

Of course, also some examples of egalitarian groups are known. Social spiders have been discussed at length elsewhere (e.g., Fronhofer, Pasurka, Mitesser, et al., 2011). Our model predicts that egalitarian animal societies evolve when resource variance is high and offspring are few. These life‐history traits are typically found in large mammals like lions (Packer et al., 2001) which do form egalitarian groups.

All these examples show that global patterns of the occurrence of eusocial and cooperatively breeding groups in natural arthropod and vertebrate systems can, at least tentatively, be explained by the above presented model, specifically by the influence of resource variance and life‐history parameters (offspring cost and number), despite its great simplicity and caveats.

## 4 CONCLUSIONS

In egalitarian as well as in eusocial groups, pooling of resources reduces the risk of starvation. In eusocial groups, it has the additional effect that it may increase intra‐group variance in the amount of resources individuals may invest in reproduction. For upward convex fertility functions, eusocial groups thus out‐compete solitary individuals as well as egalitarian groups. Whenever population growth is limited by resource availability, resources will necessarily be scarce and reproductive output will be dominantly determined by the convex part of the fertility function.

We show that in situations of limited food supply risk‐sensitive group formation has the potential to lead to the evolution of cooperative breeding and eusociality (Figure 5). More importantly, risk‐sensitivity is likely important for the maintenance of eusocial groups and in the transition from small to larger groups that had previously formed due to other mechanisms. In our model, selection for increased resource‐use efficiency leads to supersaturation (Dickinson & Hatchwell, 2004) of the environment, that is, an increase in equilibrium population density (Figure 4).

Finally, our model yields some clear and testable predictions. In summary, these are (a) Eusocial groups are favored when offspring are numerous and cheap regardless of resource variance. (b) Egalitarian groups may evolve when resource variance is high and offspring are few and costly. (c) Increasing baseline mortality favors smaller eusocial groups and ultimately solitary living. (d) Eusocial groups can evolve and be maintained despite low levels of relatedness. (e) Globally, eusocial groups should be more frequent than egalitarian animal societies.

## CONFLICT OF INTEREST

None declared.

## AUTHOR CONTRIBUTIONS

All authors designed the study. E.A.F., O.M., and H.J.P. developed and analyzed the model. E.A.F. wrote the first version of the text and all authors contributed to revisions.

## DATA ACCESSIBILITY

The R‐scripts used for numerical approximations and to generate the figures are available via Zenodo (https://doi.org/10.5281/zenodo.1481657).
