# Supplementary material for: Eusociality outcompetes egalitarian and solitary strategies when resources are limited and reproduction is costly
Source: Ecol Evol. 2018 Dec 11;8(24):12953–64. doi: 10.1002/ece3.4737 (PMC6309011; doi:10.1002/ece3.4737)
Supplement: Supplementary file 1 [file ECE3-8-12953-s001.pdf]

Supplementary Material

Emanuel A. Fronhofer, Jürgen Liebig, Oliver Mitesser
and Hans Joachim Poethke:

Eusociality outcompetes egalitarian and solitary strategies when resources are limited and reproduction is costly

Evolutionary dynamics and invasibility analysis

The basic ideas of adaptive dynamics are that 1) the resident population (here: group size strategy N_R) can be assumed to be in a dynamical equilibrium when new mutants (here: strategy N_M) appear, that 2) the fate of such mutants depends on their growth rate while rare in the population, and that 3) the effect of mutants on the optimal behavior and dynamics of residents can be neglected when mutants are rare (Diekmann, 2004). Using Eqn. 8, the assumption of a dynamical equilibrium of the resident population allows to calculate the available resources per individual $\bar{x}(N_R)$ when the resident population has reached its population equilibrium. The growth rate of an indefinitely small population of mutants following strategy N_M may subsequently be calculated as the difference between their birth- and death-rate as:

$$\Delta_M = \phi(N_M, \bar{x}(N_R), \theta) - \mu(N_M, \bar{x}(N_R), \theta). \tag{A1}$$

For positive values of Δ_M the mutant population may thus increase in size. If the growth rate is negative, no invasion is possible. This allows the presentation of pairwise invasibility plots (Fig. A1). From these plots we conclude (sensu Diekmann, 2004) that there is a continuously stable strategy (Eshel and Motro, 1981) in all cases shown in Fig. A1 and that evolution will converge towards to the singular point where the lines bordering the region of mutant growth intersect (see e.g., Geritz et al., 1998).

Fig. A1 shows that the intersection point coincides with the optimal group size N_{opt} calculated using the equilibrium approach described the main text. This clear result is explained by the fact that the mean amount of resources $\bar{x}$ collected by an individual is neither dependent on its own strategy nor on

the strategy of other members of the population but only on total population size S. If S surpasses the carrying capacity K_i of a specific strategy the population size of this strategy will necessarily decrease.

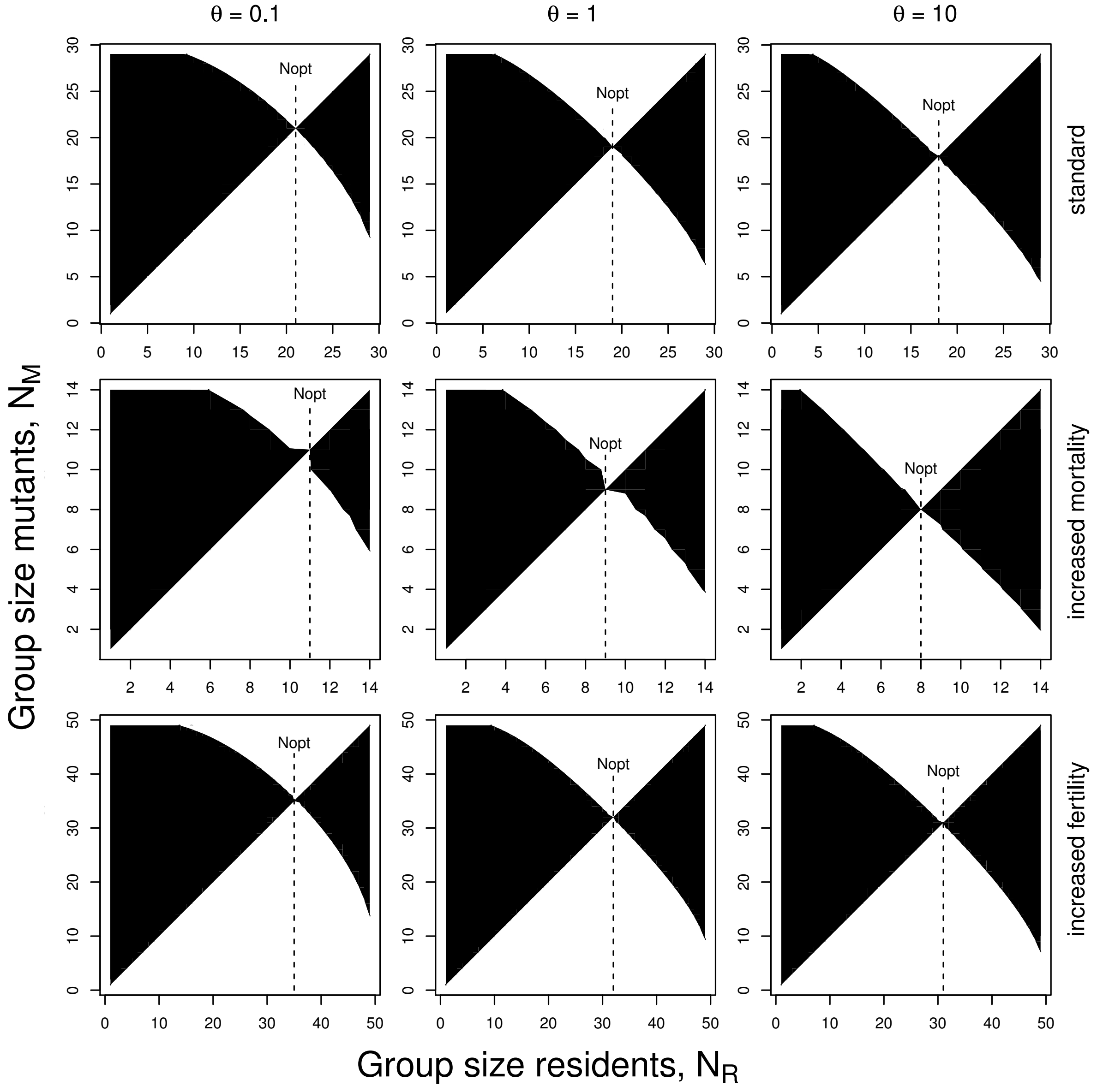

Figure A1: Pairwise invasibility plots for strategies of despotic groups. Strategies are characterized by specific group sizes. In the black area the invader population may increase in size, while it will be outcompeted by the residents in the white area. Parameter values are: standard scenario with $F_{max} = 3$ and $M_b = 0.1$; increased mortality with $F_{max} = 3$ and $M_b = 0.2$; increased fertility with $F_{max} = 5$ and $M_b = 0.1$; other parameters: $o_M = 1.0$, $c_0 = 4$. Dashed vertical lines indicate optimal group sizes (N_{opt}) calculated by the equilibrium approach in the main text.

To explicitly model the dynamics of a population consisting of individuals following different strategies N_i we use strategy dependent mortality rates $\mu(N_i, \bar{x}, \theta)$ according to Eqn. 4 and reproduction rates $\phi(N_i, \bar{x}, \theta)$ according to Eqn. 7. They allow to derive a difference equation describing the temporal

change of the population size S_i of individuals following strategy N_i:

$$S_i(t+1) = S_i(t)(1 - \mu(N_i, \bar{x}, \theta) + \phi(N_i, \bar{x}, \theta)). \tag{A2}$$

We assume a world with limited resources X where resources are replenished at each time step. Thus, the mean amount of resources $\bar{x}$ collected by an individual is dependent on total population size $\bar{x} = \frac{X}{\sum S_i}$. It is important to note in this context that $\bar{x}$ is not dependent on the specific strategy (i.e. group size N_i) of the other members of the population.

Fig. A2 shows a typical example of the resulting evolutionary dynamics. As long as the total population size is small and per capita resources are abundant, small groups perform better than large groups. This effect is caused by the limited maximum fertility F_{max} of dominant individuals. However, when populations increase and resources become limiting, the population of individuals with the optimal group size (in the specific case of Fig. A2 $N_{opt} = 19$) outcompetes all other strategies.

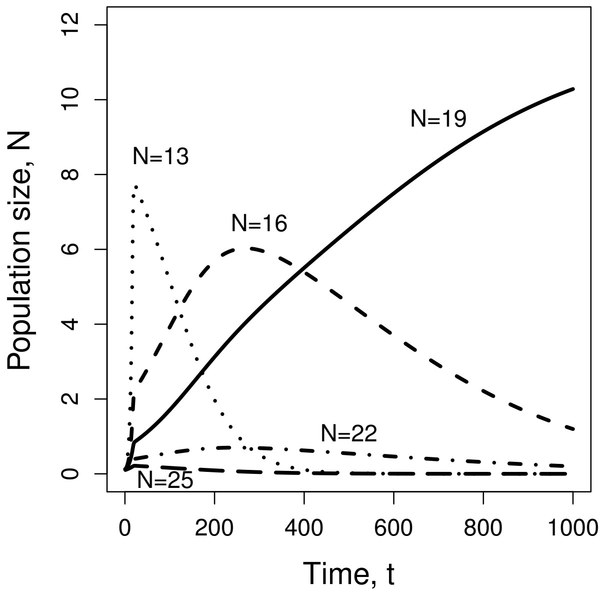

Figure A2: Example of the temporal dynamics of a population initiated at $t = 0$ with 5 different populations following the strategies $N_i = 13$, 16, 19, 22 and 25. All populations were initiated with population size $S_i(0) = 0.1$. Numerical iteration of Eqn. A2 with parameter values as in Fig. A1 (standard scenario): $F_{max} = 3$, $M_b = 0.1$, $o_M = 1.0$, $c_0 = 4$, $\theta = 1$, total amount of resources available per time step $X = 20$.

Literature cited

Diekmann, O. 2004. A beginner's guide to adaptive dynamics. Mathematical Modelling Of Population Dynamics – Banach Center Publications 63:47–86.

Eshel, I., and U. Motro. 1981. Kin selection and strong evolutionary stability of mutual help. Theor. Popul. Biol. 19:420–433.

Geritz, S. A. H., E. Kisdi, G. Meszena, and J. A. J. Metz. 1998. Evolutionarily singular strategies and the adaptive growth and branching of the evolutionary tree. Evol. Ecol. 12:35–57.
